# Reliability of movement control tests on the cervical spine

**DOI:** 10.1186/1471-2474-15-402

**Published:** 2014-11-29

**Authors:** Maja Patroncini, Susanne Hannig, André Meichtry, Hannu Luomajoki

**Affiliations:** Kantonsspital Winterthur, Brauerstrasse 15, 8401 Winterthur, Switzerland; Fetzer + Pfund. Physiotherapie. Bewegung. Training, Lindauerstrasse 112, 87439 Kempten, Germany; Zurich University of Applied Sciences, School of health professions, Institute of Physiotherapy, Technikumstrasse 70, 8400 Winterthur, Switzerland

**Keywords:** Movement control impairment, Neck pain, Reliability, Cervical spine

## Abstract

**Background:**

Movement control impairment reduces active control of movement. Patients with this might form an important subgroup among patients with mechanical cervical pain. Diagnosis is based on the observation of active movement tests. Although widely used clinically, few studies have been performed to determine the reliability of a test battery. The aim of this study was to determine the inter-tester reliability of movement control impairment [MCI] tests on the cervical spine.

**Methods:**

Forty-five subjects (31 patients with neck pain, 14 healthy controls) were videotaped while performing a standardized test battery consisting of 13 tests of active movement control. Using observation, two experienced physiotherapists independently rated test performances as correct or incorrect. One of them was blinded to all other patient information and both to each other. Kappa coefficients and 95% confidence intervals [CI] for inter-tester results were calculated.

**Results:**

The kappa values for inter-tester reliability ranged in from 0.47-1.0 of the 13 tests, 2 demonstrated perfect reliability (k = 1.0), 4 excellent (k 0.81-0.99), 6 substantial (k 0.61-0.8) and 1 good (k 0.41-0.6).

**Conclusions:**

The physiotherapists were able reliably rate the majority the tests in this series of motor control tasks. There have been studies performed describing the assessment and treatment of movement control impairment problems and low back pain. However, no study has involved the assessment of the cervical dysfunction subgroup. This study presents a reliable test battery, for clinical use, to perform more specific examination of this subgroup.

**Electronic supplementary material:**

The online version of this article (doi:10.1186/1471-2474-15-402) contains supplementary material, which is available to authorized users.

## Background

Neck pain is a common and growing health problem, with a twelve-month prevalence of between 12.1% and 71.5% [1–3]. The discussion on the causes of neck pain is controversial. The causes of idiopathic long-term neck pain and traumatic neck pain, especially, are assumed as being multifactorial [1].

Movement control appears to be an important subject in the assessment and treatment of patients with neck pain. Deficient movement control, known as movement control impairment [MCI], is defined as impaired active movement control during functional activities [4]. With regard to the cervical spine, the patient is unable to control the cervical spine during active movement. Different synonyms exist for movement control impairment: movement control dysfunction, movement system impairment or motor control impairment. Clinical instability and segmental instability can also be used as synonyms [5]. Because of deficient control of active movements lesions of the affected structures and pain can occur. MCI, in contrast to movement impairment [MI], is not marked by pain provoking restricted movement. Patients with MI typically suffer from painful restricted movement. Patients with MCI describe their problems in specific postural static positions or during ongoing unidirectional activities. In many cases, the problem is postural or ergonomic.

The subject of movement control has a long history of discussion in research literature. More recent research shows a correlation between movement control deficiency and previous or actually pain [6–10].

Several studies have assessed tests of movement control impairment of the lumbar spine [11–15]. Luomajoki and colleagues [12–14] showed good reliability and validity of movement control tests on the lumbar spine. However, compared to the lumbar spine, studies on the cervical spine are still sparse.

There is no gold standard for MCI assessment of the cervical spine [16]. According to O’Leary and collegues [17], the diagnosis of movement control should be based on the visual observation of active movements and functional activities in different starting positions. Little literature exists on the assessment of MCI of the cervical spine. To date, only basic measures for quantifying head and neck movements have been investigated, using technical equipment or visual observation [18, 19]. For more advanced investigation of MCI, it is necessary to prove the reliability of tests on the cervical spine.

The aim of the present study was to:

Assess the inter-rater reliability of active movement control tests of the cervical spine

Propose a test battery which is easy and efficient to use in practice

## Methods

### Study design

An inter-tester reliability study was performed. Forty-five participants were videotaped performing a set of thirteen active movement tests on the cervical spine. The test outcomes shown in these recordings were rated as either correct or incorrect by two experienced physiotherapists independently and in random order. The standards for correct and incorrect were defined in advance with the help of two examples. The characterization of tests and correct and incorrect performances are described in Table 1. As displayed in Table 1 if one element was not performed correctly the test was evaluated as incorrect. All videos were rated by the physiotherapists within two days, at home and using their own laptop. Each test could be observed twice. The data were registered by an independent third person and prepared for analysis. Only one rater was blinded to the participants’ baseline data. The study was performed in accordance with the Declaration of Helsinki and approval from the local ethics commission (ethical commission of canton Zürich, Switzerland) was received. Written informed consent for participation in the study was obtained from all participants.

**Table 1:** **Characterization of tests**

	Characterization of tests	Correct	Not correct	Performance	Camera position
**Rotation**	“Move your head to the right and back to middle position, to the left and back to the middle. Then move your head once through the whole range without stopping in the middle position”.	Nose stays horizontal	Evasive head movement in protraction, extension/lateral flexion or flexion	Sitting	Frontal
		No lateral flexion	Non-rhythmic movement: staccato		
		Continuous movements			
**Lateral flexion**	“Decline your head to the right and back to the middle, then to the left and back to the middle. Then move once from left to the right without stopping in the middle”.	Nose stays in the middle	Rotation	Sitting	Frontal
		No rotation	Shoulder elevation		
		No shoulder elevation	Non-rhythmic movement: staccato		
		Continuous movements	Chin heading		
**Extension CTJ**	“Draw in your chin like a little nod movement and then try to look to the ceiling”.	No chin heading	Head protraction	Sitting	Lateral 90°
		Slight global extension in CTJ	Chin heading		
		No massive distinctive extension in one segment	Shoulder elevation/protraction		
**Nod movement on the wall**	“Lean against the wall and do a small nod movement (say yes) but leave the head on the wall”.	Head moves up on the wall	Head protraction	Standing	Lateral 90°
		Draw in chin	Head moves away from the wall		
		Flattening of the lordosis	Shoulder elevation/protraction		
			Inability to draw in chin		
**Upper cervical spine**	“Tilt your head to the side and rotate it then to the ceiling.”	Visible lateral flexion and rotation	Head protraction	Sitting	Frontal
		No abolishment of lateral flexion	Shoulder elevation		
			Further going movement in the cervical spine		
**Flexion/Extension full range**	“Bring your chin to the breastbone and move your head in extension (whole movement)”.	Visible expansion of mid cervical spine while flexion	Ventral head translation while flexion	Sitting	Lateral 90°
		Rotation axis in the ear	Deficient upper cervical spine flexion		
		Round movements without protraction			
**Upper body forward - backward**	“Lean forward with straight upper body. Lean your upper body back, stay sitting straight and come back”.	Minimal chin heading	Cervical or thoracic spine flexion or extension	Sitting	Lateral 90°
		No evasive movement in the thoracic spine	Shoulder elevation/protraction		
		No movement in the cervical spine			
**Bilateral shoulder elevation**	“Lift both shoulders to the ears”.	Minimal chin heading	Cervical spine protraction	Sitting	Lateral 90° and Frontal
		Symmetric shoulder elevation	Any kind of evasive movements		
**Unilateral arm flexion right and left**	“Lift your straight arm up”.	Minimal chin heading	Cervical or thoracic spine flexion or extension	Sitting	Lateral 90° from the opposite side
		No evasive movement in the thoracic spine	Shoulder elevation/protraction		
		No movement in the cervical spine	Evasive movement in head rotation		
**Arm flexion 90° with weight**	“Lift up the weight with straight arms to 90° breast height and bring the weight with straight arms back”.	Shoulders stays down	Head protraction	Sitting	Lateral 90°
		Head stays still	Chin heading		
		Straight line of vision	Extension of cervical spine		
			Shoulder elevation		
**Forward bending in Standing**	“Bend forward and straighten up again”.	Slight extension	Head protraction	Standing	Lateral 90°
		Minimal shoulder protraction	Extension of cervical spine		
		Look towards the ground			
**Neck flexion in supine position**	“Draw in your chin and lift your head off the floor”.	Round movements	Head protraction	Supine	Lateral 90°
		No tremor	Chin heading		
		No loss of upper cervical flexion	Tremor		
			Inability to lift the head		
			Inability to draw in chin		
**Pro/retraction**	“Move your chin forward and backward”.	Horizontal nose-ear line	Shoulder elevation/protraction	Sitting	Lateral 90°
		No cervical spine extension while retraction	Flexion of thoracic spine		
			Forward-backward movement of upper body		

*CTJ*:Cervicalthoracic Junction.

### Study sample

Sample size estimation was based on earlier, similar reliability studies [12, 20, 21]. Forty-five participants were included: 31 patients with idiopathic or traumatic induced neck pain and 14 healthy volunteer subjects. Inclusion criteria for patients were neck pain, but without radiculopathy or neurological signs in the upper extremity and no known structural pathology in the cervical spine. Exclusions criteria were: neck surgery, known vertebrobasilar insufficiency, any recorded malignancy or restricted active movement of the cervical spine. The restricted active movement was examined clinically. Table 2 outlines the parameters for free movement.

**Table 2:** **Parameters for free movement**

Movement direction	Parameter
Flexion	Minimum 50°
Extension	Minimum 60°
Rotation	Minimum 80° in each direction
Lateral flexion	Minimum 45° in each direction

The inclusion criteria for healthy subjects were no neck pain and free range of motion of the cervical spine, assessed according to the parameters in Table 2. All participants were required to speak German, in order to complete the Neck Disability Index questionnaire (NDI) [22] and to follow the test instructions.

### Test protocol

**Figure 1:**
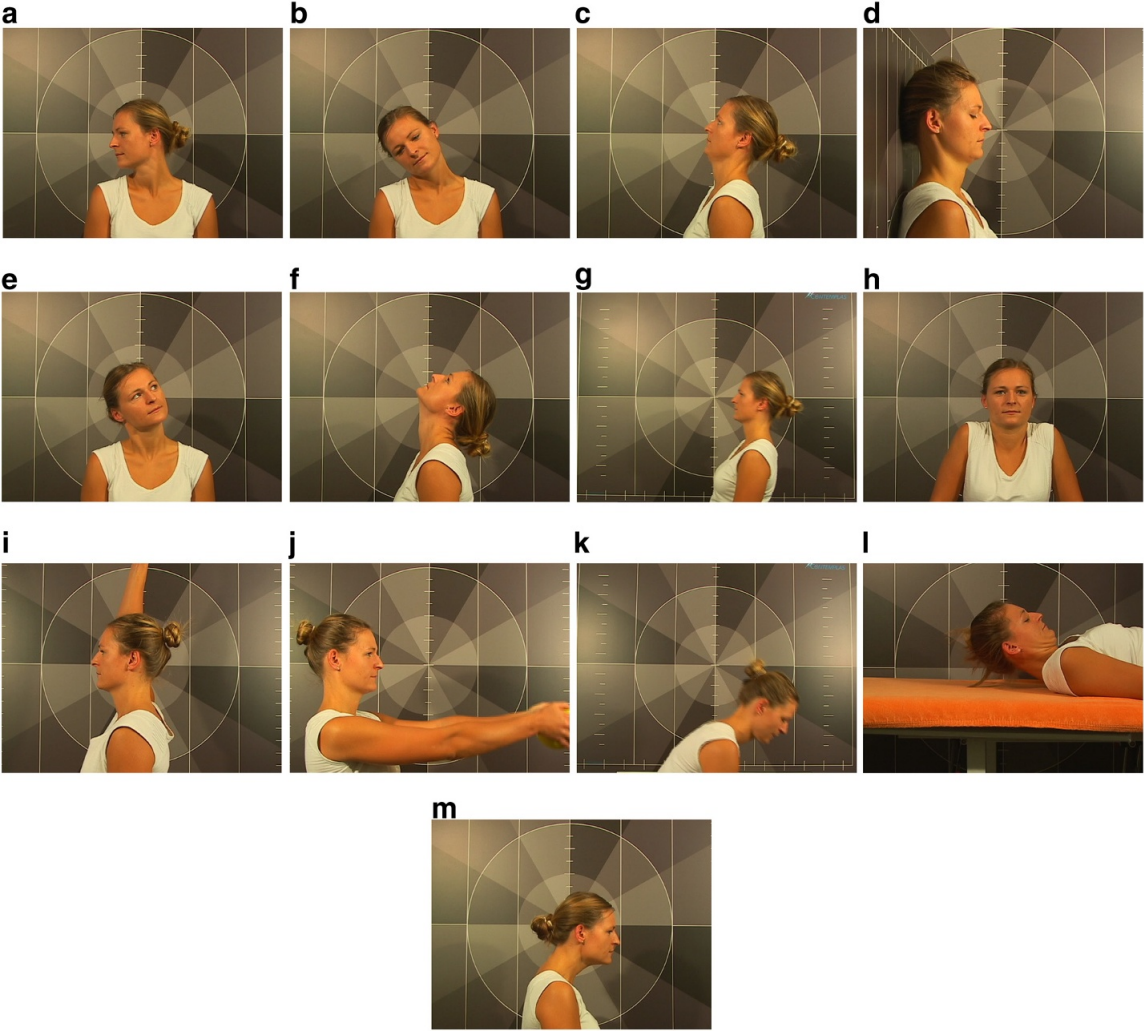
**The movement control tests used in the study. a**. Rotation. **b**. Lateral flexion. **c**. Extension CTJ. **d**. Nod movement on the wall. **e**. Upper cervical spine. **f**. Flexion/Extension full range. **g**. Upper body forward - backward. **h**. Bilateral shoulder elevation. **i**. Unilateral arm flexion. **j**. Arm flexion 90° with weight. **k**. Forward bending in standing. **l**. Neck flexion in supine position. **m**. Pro/Retraction.

Thirteen active tests were chosen to evaluate the movement control of the cervical spine (Figure 1a-1m).

The test selection was based on descriptions of Sahrmann [9, 23], and McDonnell [10, 23] and on discussions with experienced colleagues. The selected tests needed to have the capability to be filmed, observed and to contain active movements.

**Figure 2:**
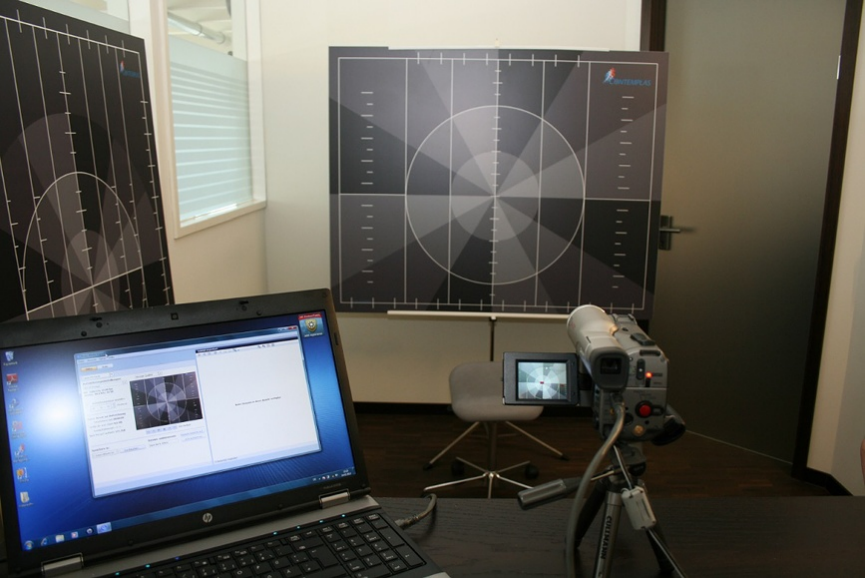
**Templo motion analysis program.**

The recordings were made using the Templo motion analysis program (http://www.contemplas.com) (Figure 2), with standardized camera locations and starting positions of the subject (Table 1). Participants received identical oral instructions. When a participant did not understand how to perform a test the explanation was repeated and, when necessary, demonstrated by the examiner. A definite recording was made of the third performed movement and this was used in the analysis.

### Statistical analysis

The data was analyzed with the statistical programs R and SSPS 19.0 for Windows. The Kappa coefficient, the 95% CI and the percentage agreement was calculated for each test. A Kappa of 1.0 indicates full agreement with no chance. Kappa values above 0.81 are considered excellent; 0.61 – 0.8 substantial; 0.41-0.6 good; 0.21-0.4 fair; and below 0.2 poor [24]. As in earlier studies [12], the definition of substantial inter-tester reliability was a test result of Kappa above 0.6. Furthermore, the lower bound of the 95% CI should be higher than 0.4.

## Results

### Subjects

Baseline data are summarized in Table 3.

**Table 3:** **Baseline data**

	Neck pain	Healthy
Number	30	15
Female/Male	26/5	7/7
Mean Age (SD)	38.3 (11.2)	39.5 (15.9)
Mean VAS (SD)	4.3 (2.3)	
Mean NDI Score (SD)	11.2 (6.0)	1.4 (2.5)
Trauma yes/no	15/15	1/14

*NDI*: Neck Disabilty Index (0–50); *SD*: Standard Deviation; *VAS*: Visual analogue scale.

### Inter-tester reliability

**Figure 3:**
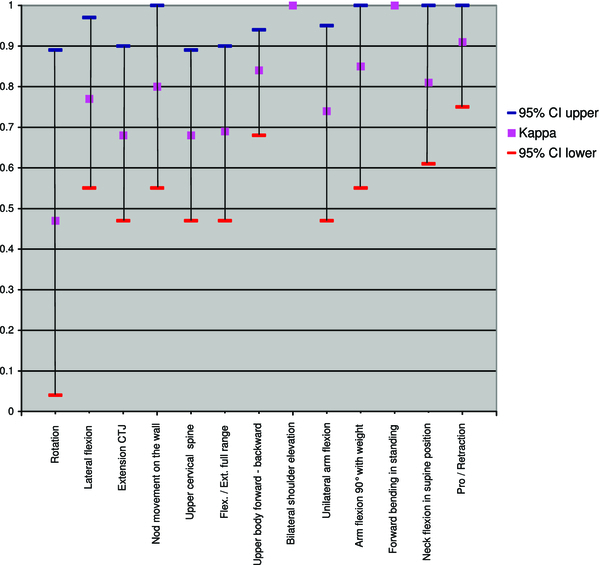
**Kappa value and 95% CI of each test.**

Table 4 shows an overview of the Kappa, the 95% CI and the percentage agreement of each movement. The Kappa values were between 0.47-1.0 (Figure 3).

**Table 4:** **Results of inter-tester reliability**

	Rotation	Lateral flexion	Extension CTJ	Nod movement on the wall	Upper cervical Rot./LF	Flex./Ext. full range	Upper body forward/backward
Kappa	0.47	0.77	0.68	0.8	0.68	0.69	0.84
95% CI	0.04-0.89	0.55-0.97	0.47-0.9	0.55-1.0	0.47-0.89	0.47-0.9	0.68-0.94
% agreement	93.3	88.3	84.4	95.5	84.5	84.4	94.5
	**Bilateral shoulder elevation**	**Unilateral arm flexion**	**Arm flexion 90° with weight**	**Forward bending in standing**	**Neck flexion in supine position**	**Pro/retraction**	
Kappa	1	0.74	0.85	1	0.81	0.91	
95% CI		0.47-0.95	0.55-1.0		0.61-1.0	0.75-1.0	
% agreement	100	97.8	97.7	100	93.3	96.3	

*CTJ*:Cervicalthoracic Junction, *Rot*.: Rotation, *LF*: Lateralflexion, Flex.: Flexion, Ext.: Extension.

The tests with highest reliability were “bilateral shoulder elevation” and “forward bending in standing” (Kappa of 1). The poorest test was “rotation”, which had Kappa below 0.6 and 95% CI of 0.04-0.89.

Data from the blinded therapist showed that, on average, the patients with neck pain performed 4.6 out of 13 tests incorrectly compared to 2.7 of the healthy subjects. This difference between the groups was highly significant (p < 0.01). Nevertheless, these data were not a subject of follow up in this study.

## Discussion

The aim of the study was to examine the inter-tester reliability of a number of movement control tests for the cervical spine. The results showed good reliability for the performed tasks, although the Kappa values varied largely (0.23 – 1.0) between the single tests. The tests for “rotatory movements” showed the lowest reliability. One reason for this may be that the tests were taped with only one camera, resulting in a potential loss of the dimensionality of the movement. The threshold of 0.6 can be considered as conservative and strict. A three-point Likert scale might have resulted in different Kappa values as found in similar studies [25]. However, we decided to use the dichotomy scale (correct/incorrect) in order to keep it simple for the clinical practice. Through the dichotomy scale, it is possible to rate the whole package as a score, where a higher score of positive tests shows a greater movement control deficit.

Only few descriptions of movement control tests for the cervical spine can be found in research literature. Most of the studies analysed a specific muscle group, one specific test or a specific clinical picture [6, 7, 26–30]. A test battery, consisting of several different tests, has not yet been described. Due to this scarcity of information, and in order to assess and identify the most effective tests, a relatively large number of tests were included. It remains arguable as to whether the tests selected by the authors are the most appropriate for movement control assessment. Further studies need to identify which combination of tests can best differentiate between patients and healthy subjects.We decided to videotape participants performing the tests to exclude all disruptive elements. This choice also allowed us to rate independently and for at least one of the physiotherapists to remain blinded to the participants. Using the reference lines (Figure 2) we created a laboratory setting. The reference lines helped to videotape in standardized starting positions. For this reason also the Kappa limit was set as high as 0.6. What is not known, however, is whether reliability would be equally as good without these lines. This question remains open and is a limitation of our study.

Different aspects led to the tests chosen in this study. The capability to film and observe was necessary in order to allow easy and efficient handling in practice. Four- point kneeling as an initial position for movement control tests is described by different authors [3, 10, 23]. But for us, it was difficult to find a standardized film position that would allow an observable evaluation and so the decision was made to exclude this position. Additionally, it was important to choose tests which offered an approach to treatment.

Because of the organisation of the study, one of the two raters was not blinded to the subjects’ diagnoses. However, this should not have diminished the assessment of reliability.

Patients with specific neck pain (ie. radiculopathy or neurological signs in the upper extremity and structural pathology in the cervical spine) were excluded. Furthermore, the group of patients showing movement impairment (ie restricted active movements), as described by O’Sullivan [4], was also excluded. Pain-free movement was a prerequisite to perform these tests. Not excluded were patients with central maladaptive pain. Participants were not examined for this. There was no differentiation between non-mechanical and mechanical neck pain, which would be important for the clinical relevance of the tests. This can be considered as a further limitation of the study.

As the inclusion criteria indicate, these tests are not relevant to patients showing specific neck pain but for patients with mechanical ischaemic pain through postural and ergonomic causes.

There were more women in the patient group than in the healthy volunteer group. This reflects the epidemiologic data, which provide higher prevalence of neck pain in women than men [1, 31]. The mean age of the patient group was 38.3 years and was in the age range with peak of prevalence for neck pain [32–34]. Mean VAS (4.3) and NDI (mean 11.2 points/50) showed that patients included in the study did not suffer intense pain and that the pain did not present a significant limitation on their daily lives. This was expected based on the inclusion and exclusion criteria. MCI is not characterized by intense pain and extreme limitation in daily life. Two participants of the healthy subjects group reported historical neck trauma. Since they hadn’t experienced a problem for many years, they were included in the healthy subjects group.

Reliable and valid tests are needed which are easy and efficient to use in clinical practice. The authors propose to use the following eight tests as a battery: extension cervicalthoracic junction [CTJ], upper body forward – backward, bilateral shoulder elevation, unilateral arm flexion, arm flexion 90° with weight, forward bending in standing, neck flexion in supine position and pro/retraction. These tests do not require any technical devices and are easy to perform. However, reference lines were used in our study and can be recommended to use in clinical setting also.

Further research should evaluate the intra-tester reliability. Two experienced manual therapists rated the data in the present study. Accordingly, it would be informative to understand how the reliability is affected when rated by less experienced manual therapists.

The significant difference between the performances of patients with neck pain versus healthy subjects shows potential for discriminative power and should be further investigated. It is also recommended to ascertain the appropriate combination of tests for optimal discriminative validity between patients and healthy controls.

## Conclusion

In the present study, patients with neck pain and healthy volunteers were videotaped performing active movement control tests of the cervical spine. The statistical analysis showed good to excellent inter-tester reliability.

Eight tests to be used as a battery are recommended. This test battery can be performed without any technical devices and is fast and efficient in clinical practice.

## Authors’ original submitted files for images

Below are the links to the authors’ original submitted files for images. Authors’ original file for figure 1 Authors’ original file for figure 2 Authors’ original file for figure 3 Authors’ original file for figure 4 Authors’ original file for figure 5 Authors’ original file for figure 6 Authors’ original file for figure 7 Authors’ original file for figure 8 Authors’ original file for figure 9 Authors’ original file for figure 10 Authors’ original file for figure 11 Authors’ original file for figure 12 Authors’ original file for figure 13 Authors’ original file for figure 14 Authors’ original file for figure 15

## Abbreviations

CI: Confidence interval
CTJ: Cervicalthoracic junction
MCI: Movement control impairment
MI: Movement impairment
NDI: Neck disability index
VAS: Visual analogue scale.
