# Supplementary figures and images for: Reliability of movement control tests on the cervical spine
Source: BMC Musculoskelet Disord. 2014 Nov 29;15:402. doi: 10.1186/1471-2474-15-402 (PMC4506480; doi:10.1186/1471-2474-15-402)

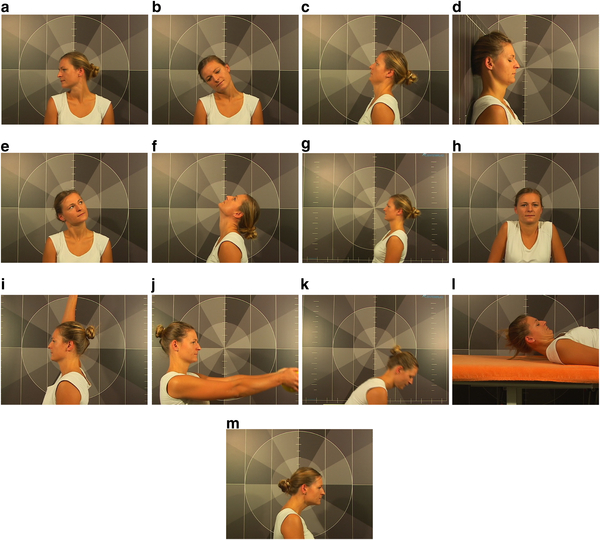

Supplement: Supplementary file 1 — Authors’ original file for figure 1 [file 12891_2014_2394_MOESM1_ESM.jpg]

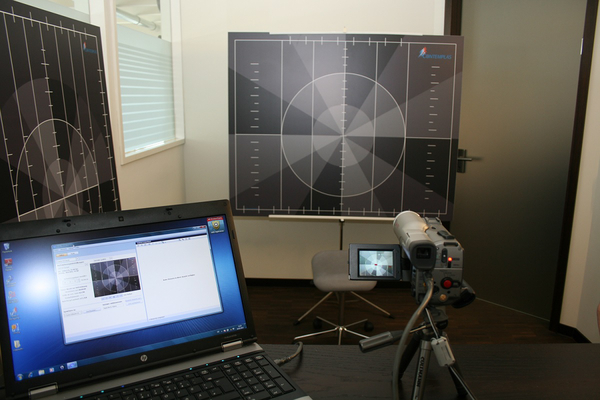

Supplement: Supplementary file 2 — Authors’ original file for figure 2 [file 12891_2014_2394_MOESM2_ESM.jpg]

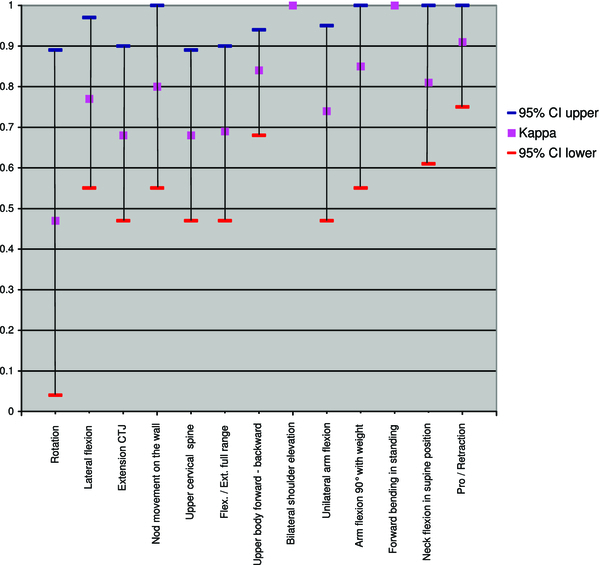

Supplement: Supplementary file 3 — Authors’ original file for figure 3 [file 12891_2014_2394_MOESM3_ESM.jpg]

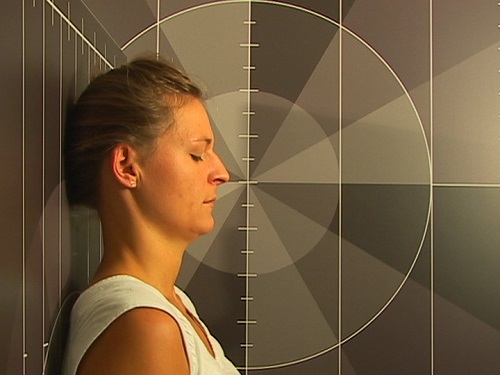

Supplement: Supplementary file 4 — Authors’ original file for figure 4 [file 12891_2014_2394_MOESM4_ESM.jpeg]

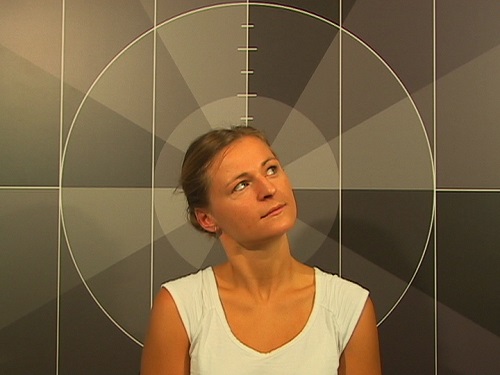

Supplement: Supplementary file 5 — Authors’ original file for figure 5 [file 12891_2014_2394_MOESM5_ESM.jpeg]

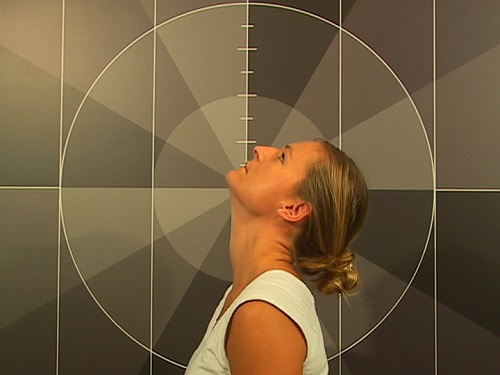

Supplement: Supplementary file 6 — Authors’ original file for figure 6 [file 12891_2014_2394_MOESM6_ESM.jpeg]

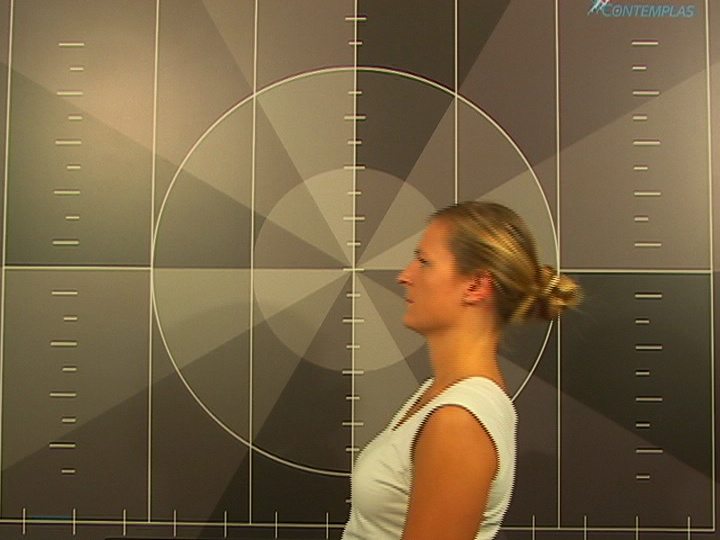

Supplement: Supplementary file 7 — Authors’ original file for figure 7 [file 12891_2014_2394_MOESM7_ESM.jpeg]

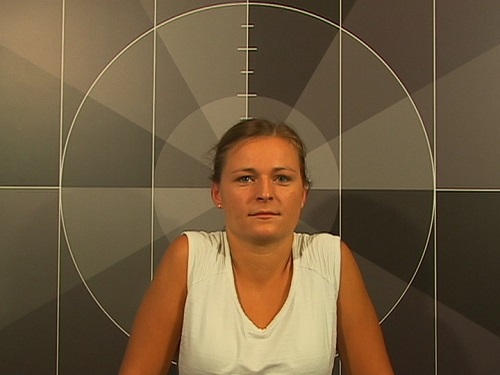

Supplement: Supplementary file 8 — Authors’ original file for figure 8 [file 12891_2014_2394_MOESM8_ESM.jpeg]

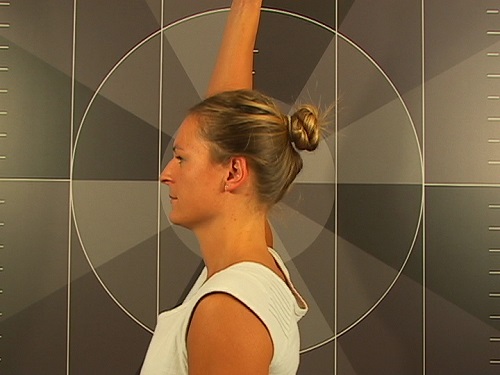

Supplement: Supplementary file 9 — Authors’ original file for figure 9 [file 12891_2014_2394_MOESM9_ESM.jpeg]

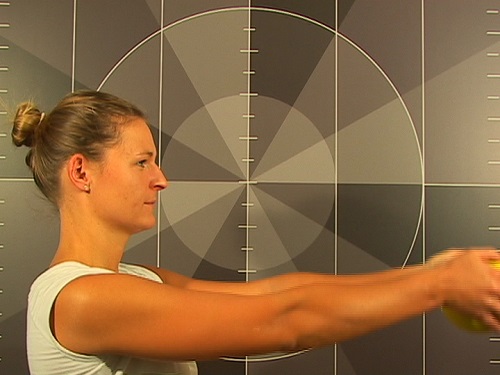

Supplement: Supplementary file 10 — Authors’ original file for figure 10 [file 12891_2014_2394_MOESM10_ESM.jpeg]

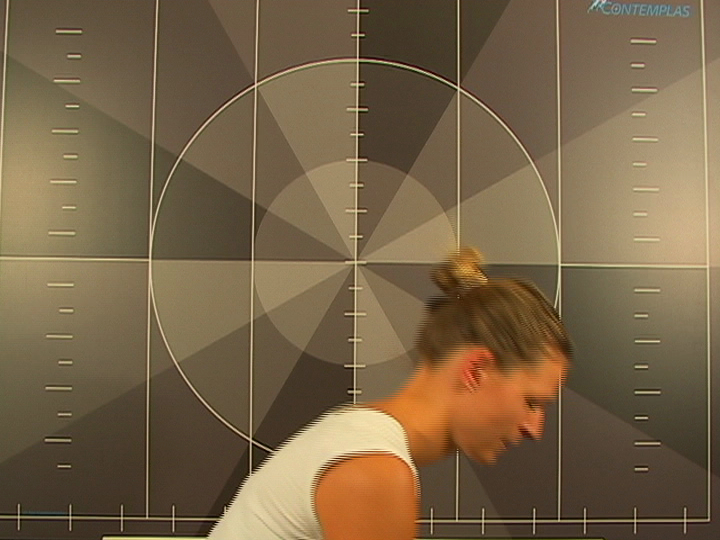

Supplement: Supplementary file 11 — Authors’ original file for figure 11 [file 12891_2014_2394_MOESM11_ESM.jpeg]

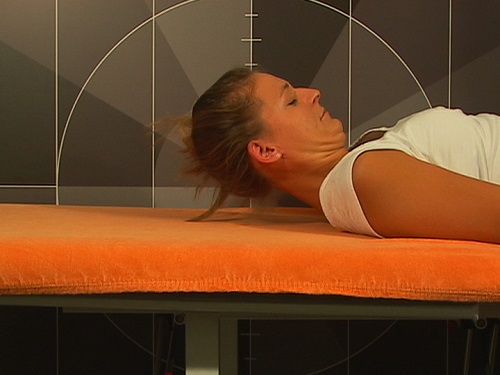

Supplement: Supplementary file 12 — Authors’ original file for figure 12 [file 12891_2014_2394_MOESM12_ESM.jpeg]

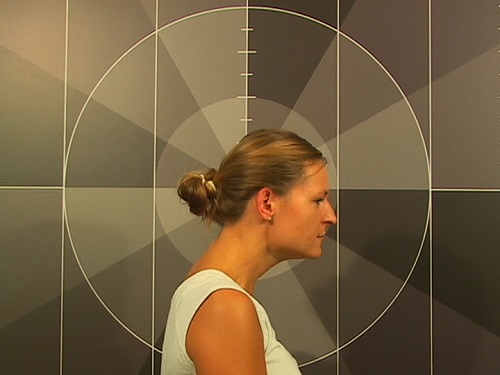

Supplement: Supplementary file 13 — Authors’ original file for figure 13 [file 12891_2014_2394_MOESM13_ESM.jpeg]

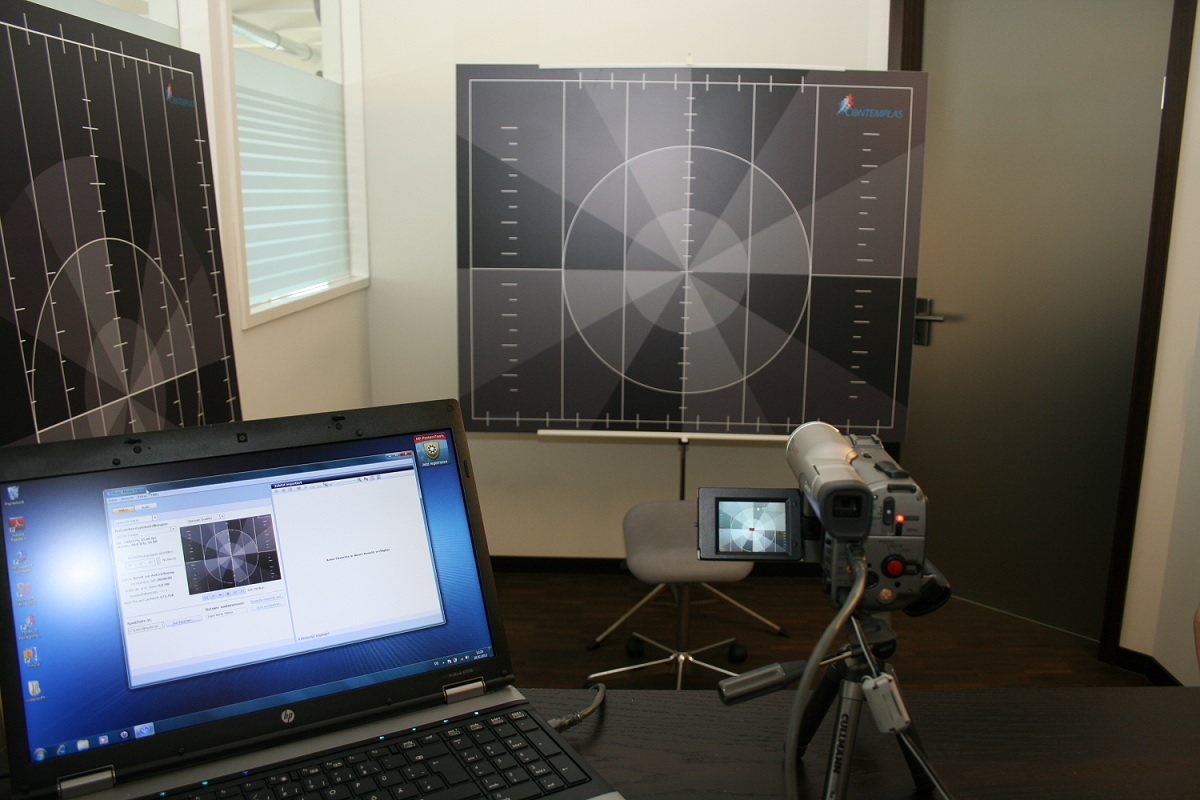

Supplement: Supplementary file 14 — Authors’ original file for figure 14 [file 12891_2014_2394_MOESM14_ESM.jpeg]

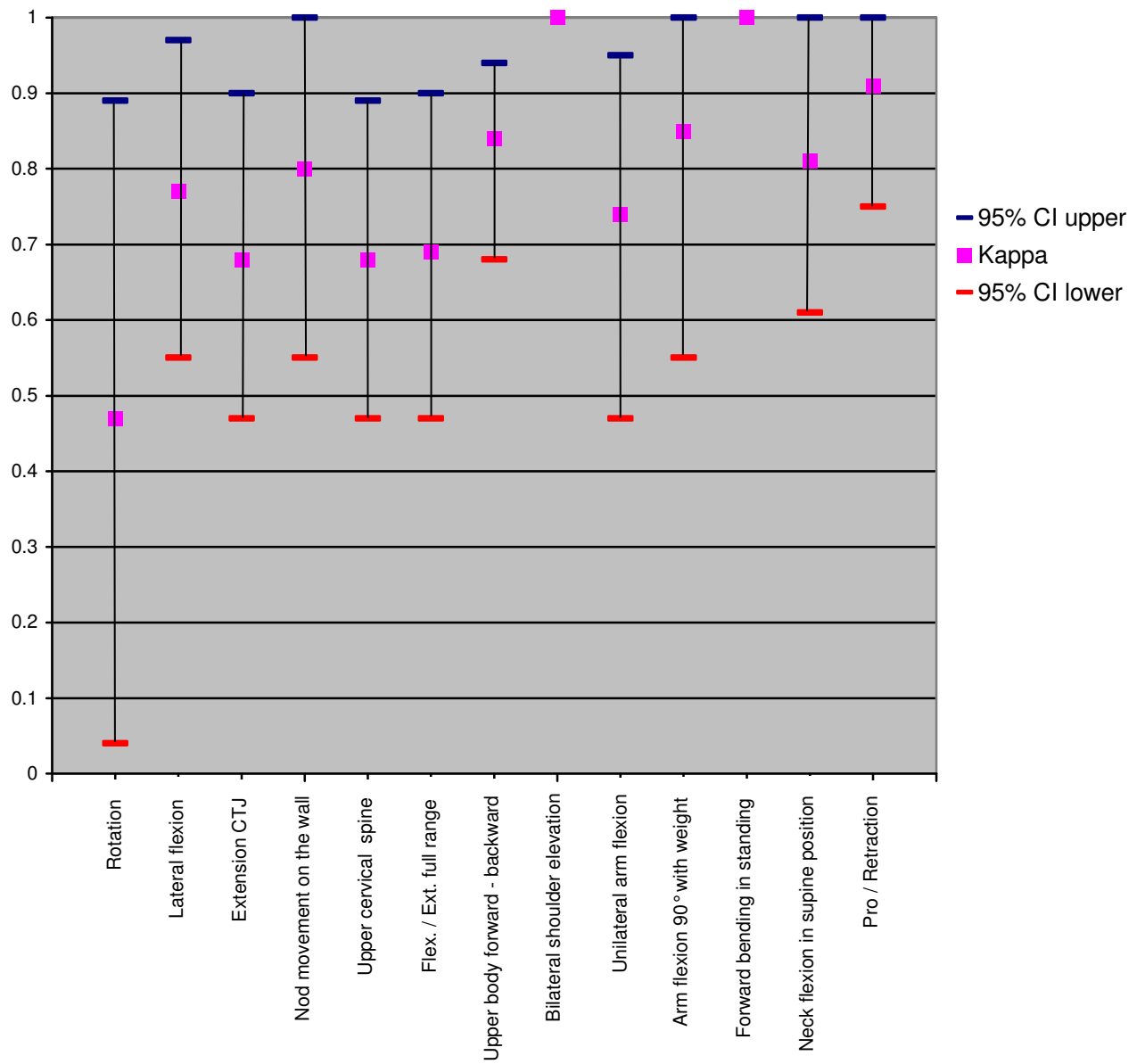

Supplement: Supplementary file 15 — Authors’ original file for figure 15 [file 12891_2014_2394_MOESM15_ESM.pdf]
